# Gender differences in the incidence of psychiatric disorders among breast cancer patients: a nationwide cohort study

**DOI:** 10.1017/S2045796025100401

**Published:** 2026-01-09

**Authors:** Dooreh Kim, Hye Sun Lee, Soyoung Jeon, Jooyoung Oh, Chang Ik Yoon

**Affiliations:** 1 Department of Surgery, Seoul St. Mary’s Hospital, College of Medicine, The Catholic University of Korea, Seoul, Republic of Korea; 2 Biostatistics Collaboration Unit, Yonsei University College of Medicine, Seoul, Republic of Korea; 3 Department of Psychiatry, Gangnam Severance Hospital, Yonsei University College of Medicine, Seoul, Republic of Korea; 4 Institute of Behavioral Sciences in Medicine, Yonsei University College of Medicine, Seoul, Republic of Korea

**Keywords:** anxiety disorder, breast cancer, depressive disorder, gender difference, mental illness, sleep disorder

## Abstract

**Aims:**

While breast cancer is rare in men, its incidence is rising, prompting more research into the mental health impacts of the disease in male patients. Anxiety, depression and sleep disorders are well-documented in women with breast cancer, but the effects on men are not as well understood, underscoring a need for gender-specific analysis.

**Methods:**

This retrospective cohort study used data from the Health Insurance Review & Assessment Service from 2009 to 2017, examining patients diagnosed with ductal carcinoma in situ or invasive breast cancer. A propensity score matching at a 5:1 ratio resulted in a sample size of 280 men and 1,400 women for analysis. The study assessed the cumulative incidence of anxiety, depression and sleep disorders, along with potential risk factors for these conditions.

**Results:**

Out of 75,936 breast cancer patients, 0.4% (281) were men. Women exhibited a significantly higher incidence of mental health conditions compared to men (*p* = 0.017), particularly in terms of anxiety. However, there were no significant gender differences in the incidence of depression or sleep disorders. Women demonstrated a higher risk of developing anxiety disorders (hazard ratio: 1.498, 95% CI: 1.057–2.123, *p* = 0.023). After adjusting for confounders, gender differences in depression and sleep disorders were not statistically significant.

**Conclusions:**

Women with breast cancer experience higher rates of anxiety disorders, while depression and sleep disorders show no gender disparity. These findings suggest that mental health care approaches should be adapted to better support men with breast cancer and address their unique mental health needs.

## Key Points

**Question.** What are the incidence rates and risk factors of depression, anxiety and sleep disorders in men and women with breast cancer?

**Findings.** In this cohort study (2009–2017), women with breast cancer had a significantly higher cumulative incidence of anxiety disorders than men. However, no significant gender differences were observed in depression or sleep disorders after adjusting for confounders.

**Meaning.** Women are more prone to anxiety, suggesting the need for gender-specific mental health strategies. However, depression and sleep disorders show no significant gender difference, highlighting the need for tailored psychiatric interventions for both genders.

**Key messages**

- The study asked whether breast cancer affects mental health conditions like anxiety, depression and sleep disorders differently in men and women.
- Women with breast cancer had a higher incidence of anxiety than men, but no gender differences were observed for depression or sleep disorders.

These findings underscore the need for gender-specific mental health care to improve outcomes for both men and women with breast cancer.

## Introduction

Male breast cancer is rare, accounting for less than 1% of all diagnosed cases. According to data from the Surveillance, Epidemiology, and End Results (Giordano *et al.*, 2004), the incidence of breast cancer in males is rising at a higher rate than that in women. In 2019, the American Cancer Society estimated that approximately 2,670 new cases of male breast cancer were diagnosed in the United States (Konduri *et al.*, 2020). This pattern has also been observed in Korea, where recent studies have reported a growing number of male breast cancer cases (Hong *et al.*, 2021; Kang *et al.*, 2022, 2023; Park *et al.*, 2024).

Despite the increasing incidence, the lack of awareness among patients, caregivers and health professionals affects this underrepresented population – this lack of awareness, coupled with treatment strategies primarily focused on women, can significantly affect the psychological well-being of male patients (Brain *et al.*, 2006). Extensive research on women with breast cancer and their psychological distress following diagnosis and treatment has revealed that cancer-related distress should be addressed with proper understanding and adequate emotional support (Irvine *et al.*, 1991; Wang *et al.*, 2020).

Mental disorders such as anxiety and depression can influence patient adherence to treatment (De Souza *et al.*, 2014; Haskins *et al.*, 2019). Breast cancer survivors often face employment and financial issues, deteriorating quality of life owing to prolonged periods of adjuvant treatment, impaired physical and cognitive functioning and fear of recurrence. Specifically, over the course of long-term follow-ups, a significant proportion of patients may be at risk of developing mental disorders (Avis *et al.*, 2015; Gøtze *et al.*, 2019; Breidenbach *et al.*, 2022).

The incidence of mental disorders typically differs between men and women (Piccinelli and Wilkinson, 2000). Symptoms of depression and anxiety are more prevalent in women, influenced by hormonal and psychosocial factors (Otten *et al.*, 2021). However, it is unclear whether males with breast cancer follow typical sex differences observed in the prevalence of mental disorders or exhibit higher rates owing to their unique situation. Although some studies on the psychological impact of male breast cancer (Weber *et al.*, 2021; Abboah-Offei *et al.*, 2024) have explored defence mechanisms and repressive coping strategies, comprehensive studies determining the overall prevalence of mental disorders in this population remain limited. Given the specific needs and potential vulnerability of males with breast cancer, further research in this area is warranted.

This study primarily aimed to investigate the incidence and patterns of psychiatric disorders, specifically depression, anxiety and sleep disorders, between males and females with breast cancer following diagnosis. Secondarily, we sought to identify sex-related differences and risk factors for these psychiatric disorders. Understanding these differences is particularly important because males with breast cancer may encounter distinct psychosocial challenges and healthcare barriers that are not yet well documented in the literature.

## Methods

**Figure 1.**
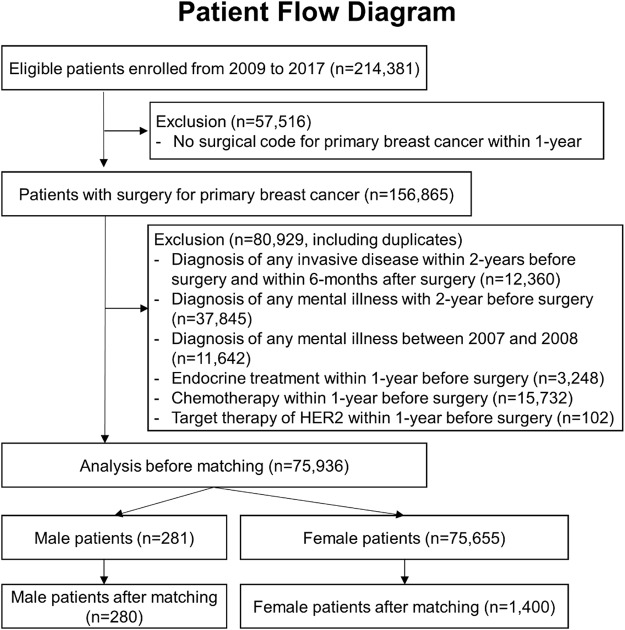
Flow diagram of patients enrolled in the retrospective cohort study.

Between 2009 and 2017, patients diagnosed with ductal carcinoma in situ or invasive breast cancer were identified using Health Insurance Review & Assessment Service claims data. Patients without a surgical behaviour code (Supplementary Table 1) within 1 year of diagnosis were categorized as having metastatic or de novo stage disease and were excluded from the analysis. A 2-year wash-out period was implemented for both breast cancer and mental illness to avoid confounding by prior diagnoses. To exclude patients undergoing primary systemic therapy or palliative treatment, any treatment administered within 1 year before surgery was not considered (Supplementary Fig. 1). Treatment modalities, including endocrine therapy, chemotherapy and targeted agents, as well as prescription codes, were used to classify patients’ treatment (Supplementary Table 2). Initially, 75,936 patients (281 men and 75,655 women) were eligible for analysis. Following a 5:1 propensity score matching, the study included 1,400 women and 280 men (Fig. 1).

This study aimed to determine the cumulative incidence of mental illness in men and women diagnosed with breast cancer. Mental illness was broadly classified into three main categories: anxiety, depression and sleep disorders, with detailed diagnostic classification provided in Supplementary Table 3. This study was also designed to investigate the risk factors associated with the development of mental illnesses. Comorbidities were assessed using the Charlson Comorbidity Index (CCI), which encompasses a range of clinically relevant chronic conditions that impact physical and mental health outcomes (De Groot *et al.*, 2003; Austin *et al.*, 2015). All components of the CCI were included in the analysis to comprehensively adjust for patients’ baseline comorbidity burden (Supplementary Table 4) (Quan *et al.*, 2005). These comorbidities were identified based on relevant diagnostic codes recorded within 2 years prior to the enrolment date.

Statistical analyses were conducted to compare baseline demographics and clinical characteristics between the two groups using *t*-tests and chi-square tests. The cumulative incidence rates of mental illness in both groups were depicted using Kaplan–Meier curves and compared using the log-rank test. Cox proportional hazard models were employed to estimate hazard ratios (HRs) and their corresponding 95% confidence intervals (CIs) were used to assess the occurrence of mental illnesses, adjusting for confounding variables. Statistical significance was set at a two-sided *p*-value of <0.05. Randomization was performed using an algorithm in SAS software (version 9.4; SAS Institute, Cary, NC, USA). To reduce bias, propensity scores were estimated and used to match male to female patients. These propensity scores were calculated for each patient using logistic regression analysis, incorporating variables such as age, type of adjuvant treatment, and comorbidities. Propensity score matching was performed using a nearest-neighbour greedy algorithm (Austin, 2010). A 5:1 propensity score matching was conducted to optimize the sample size while minimizing potential bias in the estimated outcomes, as increasing the number of controls per case yields diminishing returns in statistical power. This study was approved by our local IRB (No. KC23RISI0200).

## Results

### Patient characteristics

Between 2009 and 2017, 0.4% (281/75,936) of all patients diagnosed with breast cancer were men (Table 1). Before matching, male patients were older at diagnosis, had higher CCI scores and had fewer mental illnesses than female patients. Overall, 33% of all breast cancer patients, regardless of sex, experienced a mental illness following diagnosis. Anxiety disorders were significantly more prevalent in female patients than in their male counterparts; however, no significant sex differences were observed for depression and sleep disorders. Consistent with findings from previous studies, men received more endocrine treatment, indicating a higher prevalence of hormone receptor-positive disease, whereas the rates of chemotherapy were similar. After adjusting for age, type of surgery, treatment modality, and CCI score, the age distribution across decades was similar between the two groups.

**Table 1.** Comparison of clinical characteristics of patients with breast cancer according to sex

	Before matching			After matching		
	Male, *n* = 281 (%)	Female, *n* = 75,655 (%)	*p*-Value	Male, *n* = 280 (%)	Female, *n* = 1,400 (%)	*p*-Value
**Age (year)**			<0.0001			0.9999
**10–19**	0(0.00)	27(0.04)		4(1.43)	15(1.07)	
**20–29**	4(1.42)	882(1.17)		13(4.64)	65(4.64)	
**30–39**	13(4.63)	8,763(11.58)		56(20.00)	283(20.21)	
**40–49**	56(19.93)	29,657(39.20)		61(21.79)	307(21.93)	
**50-59**	61(21.71)	22,004(29.08)		70(25.00)	350(25.00)	
**60–69**	70(24.91)	9,774(12.92)		61(21.79)	309(22.07)	
**70–79**	61(21.71)	3,928(5.19)		13(4.64)	61(4.36)	
**80+**	14(4.98)	601(0.79)		2(0.71)	10(0.71)	
**CCI (Weight number, mean ± SD)**	4.594 ± 2.742	3.796 ± 2.193	<0.0001	4.579 ± 2.735	4.541 ± 2.729	0.8354
**Chemotherapy**			0.6464			0.9648
**Not done**	119(42.35)	33,068(43.71)		119(42.50)	597(42.64)	
**Done**	162(57.65)	42,587(56.29)		161(57.50)	803(57.36)	
**Endocrine therapy**			<0.0001			0.845
**Not done**	23(8.19)	18,784(24.83)		23(8.21)	120(8.57)	
**Done**	258(91.81)	56,871(75.17)		257(91.79)	1,280(91.43)	
**HER2-target therapy**			0.0008			0.6676
**Not done**	264(93.95)	66,054(87.31)		263(93.93)	1,324(94.57)	
**Done**	17(6.05)	9,601(12.69)		17(6.07)	76(5.43)	
**Mental illness**			0.0024			0.0126
**No**	211(75.09)	50,326(66.52)		210(75.00)	944(67.43)	
**Yes**	70(24.91)	25,329(33.48)		70(25.00)	456(32.57)	
**Anxiety disorder**			0.0057			0.0235
**No**	245(87.19)	61,024(80.66)		244(87.14)	1,141(81.50)	
**Yes**	36(12.81)	14,631(19.34)		36(12.86)	259(18.50)	
**Depressive disorder**			0.2634			0.2333
**No**	243(86.48)	63,572(84.03)		242(86.43)	1,170(83.57)	
**Yes**	38(13.52)	12,083(15.97)		38(13.57)	230(16.43)	
**Sleep disorder**			0.0777			0.1067
**No**	254(90.39)	65,689(86.83)		253(90.36)	1,216(86.86)	
**Yes**	27(9.61)	9,966(13.17)		27(9.64)	184(13.14)	

CCI, Charlson Comorbidity Index; SD, standard deviation.

### Overall incidence of anxiety, depression and sleep disorders

**Figure 2.**
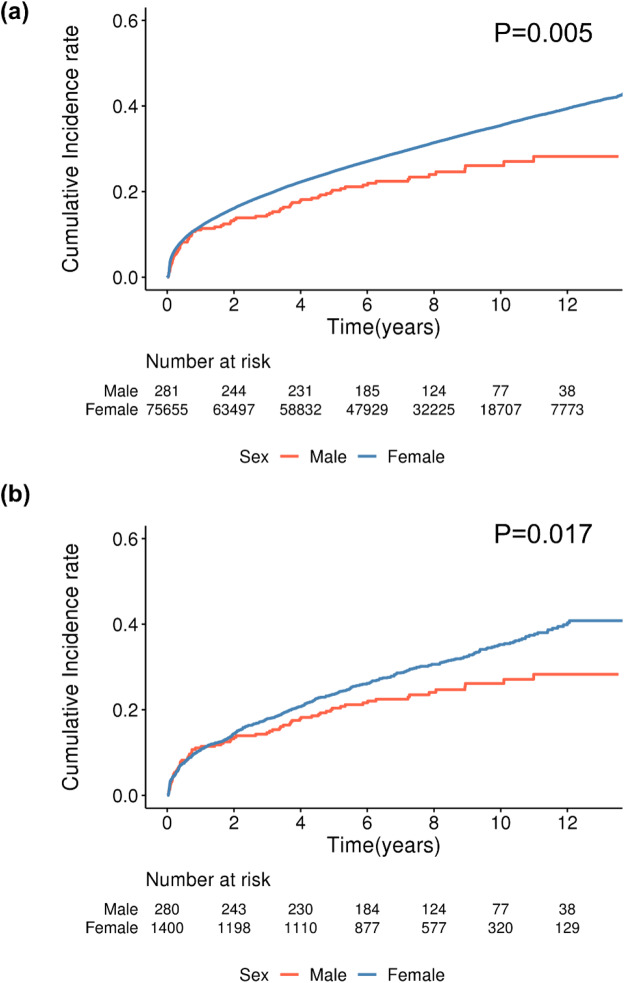
Kaplan–Meier analysis of the incidence of mental illness in breast cancer patients according to gender (median follow-up 7.30 ± 3.86 years). Before matching, there was a significant difference in the incidence of mental illnesses in women compared to men (a: male, *n* = 281; female, *n* = 75,655; *p* = 0.005; log-rank test). After matching, the incidence of mental illnesses in women was significantly higher than that in men (b: male, *n* = 280; female, *n* = 1,400; *p* = 0.017; log-rank test).

**Figure 3.**
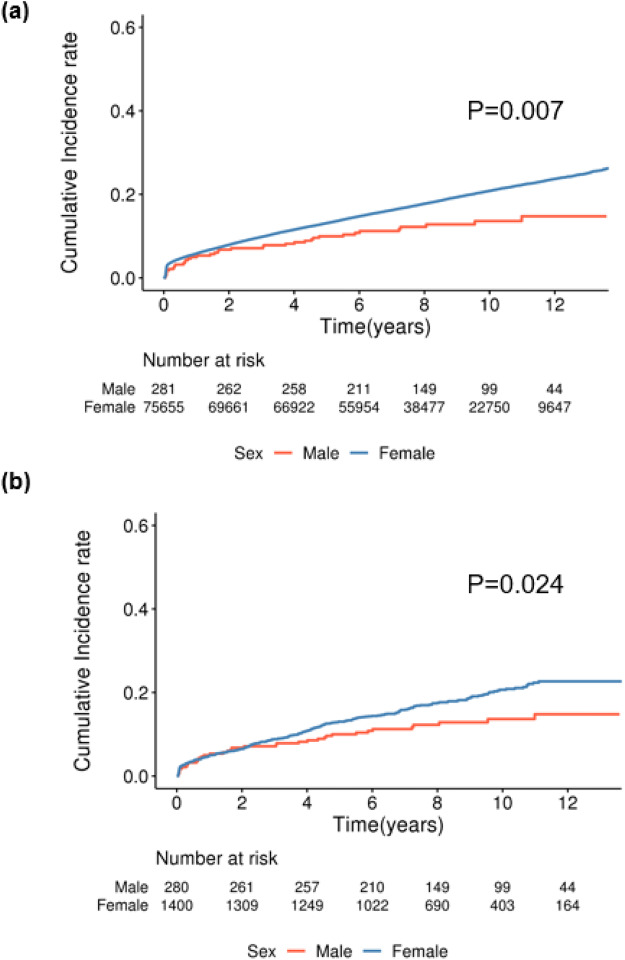
Kaplan–Meier analysis of the incidence of anxiety disorders in patients with breast cancer according to sex (median follow-up, 8.09 ± 3.42 years). Before matching, a significant difference was observed in the incidence of anxiety disorders between females and males (a: male, *n* = 281; female, *n* = 75,655; *p* = 0.007; log-rank test). After matching, the incidence of anxiety disorders in females was significantly higher than that in males (b: male, *n* = 280; female, *n* = 1,400; *p* = 0.024; log-rank test).

Before matching, there was a significant difference in the overall incidence of mental disorders between females and males. Even after matching, the incidence of mental disorders remained significantly higher in women than in men (Fig. 2a: men, *n* = 281; women, *n* = 75,655; *p* = 0.005; Fig. 2b: men, *n* = 280; women, *n* = 1,400; *p* = 0.017; log-rank test). The sex disparity was particularly evident and statistically significant for the development of anxiety disorders (Fig. 3a: before matching, *p* = 0.007; Fig. 3b: after matching, *p* = 0.024; log-rank test). However, no significant sex differences were observed for the incidence of sleep or depressive disorders, irrespective of adjuvant treatment (Supplementary Figs. 2 and 3).

### Hazard rate analysis

**Figure 4.**
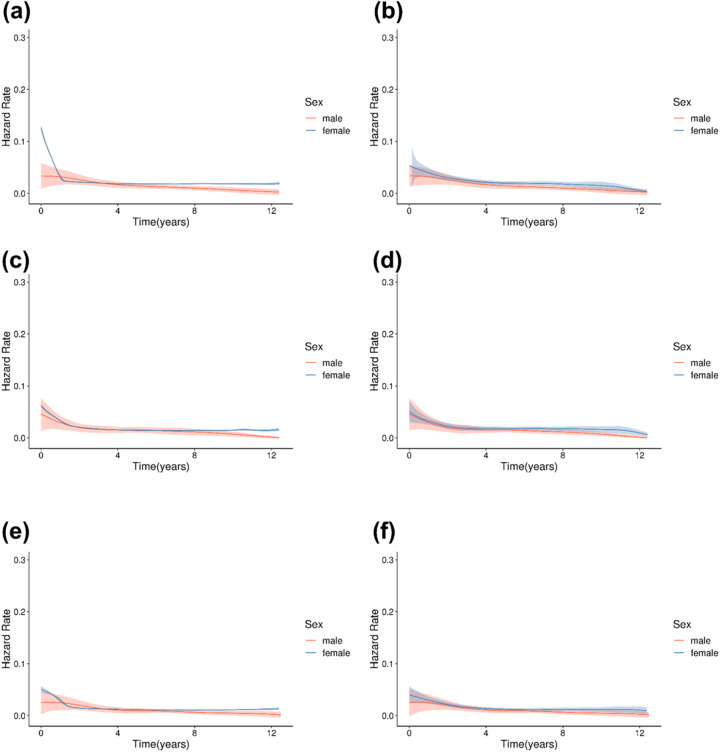
Annual hazard rate of the incidence of mental illnesses in patients with breast cancer according to sex (a) before matching, anxiety disorders; (b) after matching, anxiety disorders; (c) before matching, depressive disorders; (d) after matching, depressive disorders; (e) before matching, sleep disorders; (f) after matching, sleep disorders.

Examining the hazard rate plot depicting the incidence of mental illnesses over time, expressed as HRs, revealed that female patients initially exhibit a higher risk of mental illness (Fig. 4). However, this risk gradually declined and converged with that of male patients over time. Contrary to the common assumption that women are more vulnerable to mental disorders, the HRs for depressive and sleep disorders were similar between women and men.

### Before-matching analysis

A Cox proportional hazards regression model was used for both the before- and after-matching cohorts. Before matching, both sex and age were significant in the univariate and multivariate models. In the univariate model, female sex was associated with a HR of 1.398 (Table 2; 95% CI, 1.106–1.767; *p* = 0.005). In the multivariate model, after adjusting for other variables, the HR for female sex increased to 1.549 (95% CI, 1.225–1.958; *p* < 0.001). Additionally, age and CCI score were significantly associated with the development of a mental illness. For every one-unit increase in the CCI score, the HR increased by 1.048 and 1.036 in the univariate and multivariate analyses, respectively. However, endocrine and targeted therapies became insignificant after adjusting for other variables.

**Table 2.** Risk of developing mental illnesses based on analyses using Cox proportional hazard models

	Before matching		After matching		Before matching		After matching	
	Univariate analysis		Univariate analysis		Multivariate analysis		Multivariate analysis	
	HR (95% CIs)	*p-*Value	HR (95% CIs)	*p*-Value	HR (95% CIs)	*p*-Value	HR (95% CIs)	*p-*Value
**Age (per 10 years)**	1.079 (1.068 − 1.091)	<0.0001	1.112 (1.044–1.183)	0.0009	1.078 (1.066–1.090)	<0.0001	1.129 (1.055–1.209)	0.0004
**Sex**		0.0051		0.0177		0.0003		0.0165
**Male**	Reference		Reference		Reference		Reference	
**Female**	1.398 (1.106–1.767)		1.356 (1.054–1.744)		1.549 (1.225–1.958)		1.361 (1.058–1.750)	
**CCI (Weight number)**	1.048 (1.042–1.053)	<0.0001	1.055 (1.024 − 1.086)	0.0009	1.036 (1.031–1.042)	<0.0001	1.045 (1.014–1.077)	0.0039
**Chemotherapy**		<0.0001		0.0656		<0.0001		0.0609
**Not done**	Reference		Reference		Reference		Reference	
**Done**	1.322 (1.289–1.356)		1.179 (0.989–1.404)		1.327 (1.292–1.363)		1.192 (0.992–1.432)	
**Endocrine therapy**		0.0624		0.1336		0.2724		0.0573
**Not done**	Reference		Reference		Reference		Reference	
**Done**	0.973 (0.946–1.001)		1.287 (0.925–1.791)		1.016 (0.987–1.046)		1.390 (0.990–1.952)	
**HER2–target therapy**		<0.0001		0.2312		0.3264		0.1701
**Not done**	Reference		Reference		Reference		Reference	
**Done**	1.175 (1.133–1.217)		1.244 (0.870–1.779)		1.019 (0.981–1.058)		1.294 (0.895–1.871)	

CCI, Charlson Comorbidity Index.

### After-matching analysis

Analysis of the after-matching cohort showed that female sex remained a risk factor for the occurrence of mental disorders. Moreover, for every 10-year increase in age, the HR increased by 1.112 and 1.129 in the univariate and multivariate analyses, respectively (Table 2). This trend was consistent with the finding for the CCI score. However, none of the treatment modalities, especially chemotherapy, significantly affected the development of mental disorders (Table 2).

When mental illnesses were analysed by separate disease entities, men and women showed similar risk of developing the mental illness, except for anxiety disorders. Female sex was the only statistically significant risk factor (49% higher risk than males) for developing anxiety disorder (Supplementary Tables 5–7, Supplementary Table 5; HR 1.498, 95% CI 1.057–2.123, *p* = 0.023). Age and CCI score were significant risk factors for depressive disorders (Supplementary Table 6), whereas only CCI score was an independent risk factor for sleep disorders (Supplementary Table 7). The pronounced sex disparity in the overall incidence of mental illnesses was primarily due to the higher prevalence of anxiety disorders among female patients.

## Discussion

Our study highlighted significant sex differences in the incidence of depression, anxiety and sleep disorders among patients with breast cancer, particularly owing to the higher prevalence of anxiety disorders in women. This finding aligns with those of existing studies (Piccinelli and Wilkinson, 2000; Otten *et al.*, 2021), which have shown that women are generally more susceptible to emotional problems owing to various hormonal and psychosocial factors.

Considering the frequent comorbidity of anxiety and depression (Groen *et al.*, 2020; Wang *et al.*, 2023), the divergence in their prevalence between sexes observed in our study is noteworthy. While women exhibited significantly higher rates of anxiety disorders than men, differences in the incidence of depressive disorders were less pronounced. The sex-specific disparity observed in anxiety disorders, but not in depressive or sleep disorders, may be attributed to a combination of neuroendocrine, psychosocial, and behavioural mechanisms.

Hormonal influences such as fluctuations in estrogen and progesterone are known to acutely affect neural circuits involved in fear and arousal, such as the amygdala and hypothalamic-pituitary-adrenal (HPA) axis (Lange and Erhardt-Lehmann, 2025), thereby amplifying anxiety responses in women, especially during reproductive transitions like menstruation, pregnancy, and menopause (Wieczorek *et al.*, 2023). Conversely, while depression is also linked to HPA axis dysfunction (Pariante and Lightman, 2008), its onset and course are more gradual and shaped by a broader range of factors, including cognitive appraisal, social support and chronic stress exposure (Monroe and Harkness, 2005). These characteristics render depressive symptoms less directly linked to hormonal fluctuations and more variable across individuals, which may explain the absence of a consistent sex difference in its incidence in our study.

Psychosocial factors could also contribute to this divergence. Women tend to adopt more emotion-focused coping strategies and may be more attuned to internal emotional states, which facilitates help-seeking behaviour and clinical recognition of anxiety symptoms. Men, in contrast, often exhibit avoidant or repressive coping styles and may underreport psychological distress due to prevailing norms of masculinity that discourage emotional vulnerability. Consequently, anxiety symptoms in men may remain unrecognized, particularly in healthcare settings that rely on self-reporting or externally visible distress. Depressive symptoms, such as persistent low mood, anhedonia and fatigue, are often more internalized and nonspecific, making them harder to detect across both sexes. Moreover, the gradual and heterogeneous nature of depressive symptom onset may obscure sex-based differences in diagnosis, especially in claims-based datasets. This could explain the less pronounced sex disparity observed in depressive disorders in our cohort.

Anxiety disorders often present with more immediate and disruptive symptoms, such as intense fear, panic and physiological arousal (He *et al.*, 2023), making them more noticeable and reportable. Conversely, depression can manifest in more subtle ways, including persistent sadness, lack of energy and loss of interest in daily activities (Kumar *et al.*, 2012). These symptoms may go unreported or unrecognized, particularly in individuals who internalize their distress or express it through behaviours such as substance use, leading to underdiagnosis or misdiagnosis (de León *et al.*, 2019). This challenge is further compounded for non-psychiatric healthcare providers, who may struggle to detect such subtle or atypical presentations.

This behavioural asymmetry is also reflected in the temporal pattern observed in our data: although women had a higher initial incidence of mental illnesses, the hazard converged over time between the sexes. This suggests that while women may experience and report psychological distress earlier, it may emerge later or in subtler forms in men, highlighting a delayed emergence rather than a true absence of distress. This may also be attributed to men’s tendency to repress their emotional distress, a behaviour often rooted in societal expectations of masculinity (Kim and Yu, 2023). As the disease progresses, men may eventually express or experience psychological distress similar to women, highlighting the need for ongoing mental health support throughout the treatment continuum for both men and women.

Overall, these findings underscore the need for sex-sensitive mental health screening approaches in oncology settings. Relying solely on diagnostic incidence may underestimate the mental health burden in male patients, particularly for conditions like anxiety, which are more likely to be underreported due to sex patterns of emotional expression and help-seeking. Longitudinal and qualitative research is needed to clarify how emotional distress manifests differently across sexes and to inform tailored psychiatric interventions for breast cancer survivors.

Our findings also indicate that after adjusting for several variables, such as the CCI score, endocrine treatments and chemotherapy were not significantly associated with the development of psychiatric disorders. This is consistent with a previous finding, which revealed that endocrine treatments, including tamoxifen, do not increase the risk of depression (Oh *et al.*, 2022). Hence, apprehensions regarding depression, anxiety and sleep disorders should not deter men or women from pursuing essential treatments.

The limitations of this study include the lack of data from medical records and the inability to verify individual information. Since this study used big data based on operational definitions, there may be an overestimation or underestimation of mental illness diagnoses. While a broad spectrum of anxiety and depressive disorders was considered, more detailed diagnostic information was unavailable. Moreover, despite the use of propensity score matching to adjust for key confounders, residual confounding may still exist. Specifically, unmeasured variables such as personal coping styles, prior mental health status social support networks, and other psychosocial factors could have impacted the observed mental health outcomes. This represents an inherent limitation of our retrospective study design.

Overall, our study underscores the importance of sex-sensitive psychiatric interventions for males with breast cancer. Given the unique challenges and tendency to underreport symptoms in this population, healthcare practitioners should adopt more proactive screening measures to ensure the timely identification and management of mental health issues. Enhancing awareness among healthcare providers and developing targeted guidelines for male breast cancer care could improve both quality of life and overall patient outcomes. Future research should focus on comprehensive and prospective clinical trials to better understand the evolving mental health needs of this population and to evaluate the clinical effectiveness of targeted, sex-sensitive interventions that can be translated into routine clinical practice.

## Supporting information

10.1017/S2045796025100401.sm001 Kim et al. supplementary material Kim et al. supplementary material

## Data Availability

The data used in this study are not publicly available, as access to the database is restricted after a certain period and requires a fee to unlock.

## Supplementary material

The supplementary material for this article can be found at https://doi.org/10.1017/S2045796025100401.
