# Gender differences in the incidence of psychiatric disorders among breast cancer patients: a nationwide cohort study – CORRIGENDUM

**DOI:** 10.1017/S2045796026100444

**Published:** 2026-01-30

**Authors:** Dooreh Kim, Hye Sun Lee, Soyoung Jeon, Jooyoung Oh, Chang Ik Yoon

The Author contributions and Financial support sections should read as follows.

**Author contributions**. Chang Ik Yoon and Jooyoung Oh (ojuojuoju@yuhs.ac) contributed equally to this work and should be considered co-corresponding authors.

**Funding statement.** This work was supported by the Korea Medical Device Development Fund grant funded by the Korea government (the Ministry of Science and ICT, the Ministry of Trade, Industry and Energy, the Ministry of Health & Welfare, the Ministry of Food and Drug Safety) (Project Number: 1711174512, RS-2022-01140408).

The authors apologize for the error.
