# Supplementary material for: Gender differences in the incidence of psychiatric disorders among breast cancer patients: a nationwide cohort study
Source: Epidemiol Psychiatr Sci. 2026 Jan 9;35:e5. doi: 10.1017/S2045796025100401 (PMC12816929; doi:10.1017/S2045796025100401)
Supplement: Kim et al. supplementary material [file S2045796025100401sup001.docx]

**Supplementary Figure 1. Patient enroll diagram**


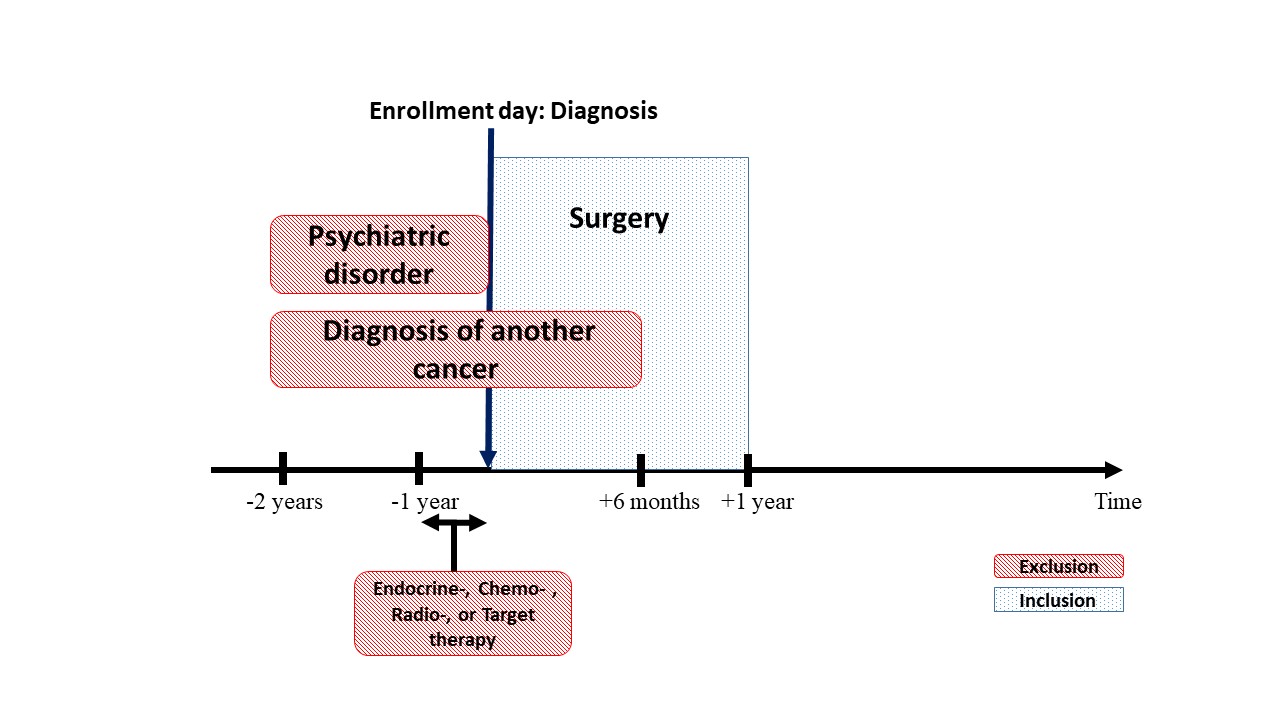


**Supplementary Figure 2. Kaplan–Meier analysis of the incidence of depressive disorder in breast cancer patients according to gender (median follow up 8.15±3.28 years). B**efore matching, there was no significant difference in the incidence of depressive disorder in women compared to men (a, male n=281, female n=75,655, p=0.275, log-rank test). After matching, there was still no statistical difference in the incidence of depressive disorder in women compared to men (b, male n=280, female n=1,400, p=0.224, log-rank test).

**
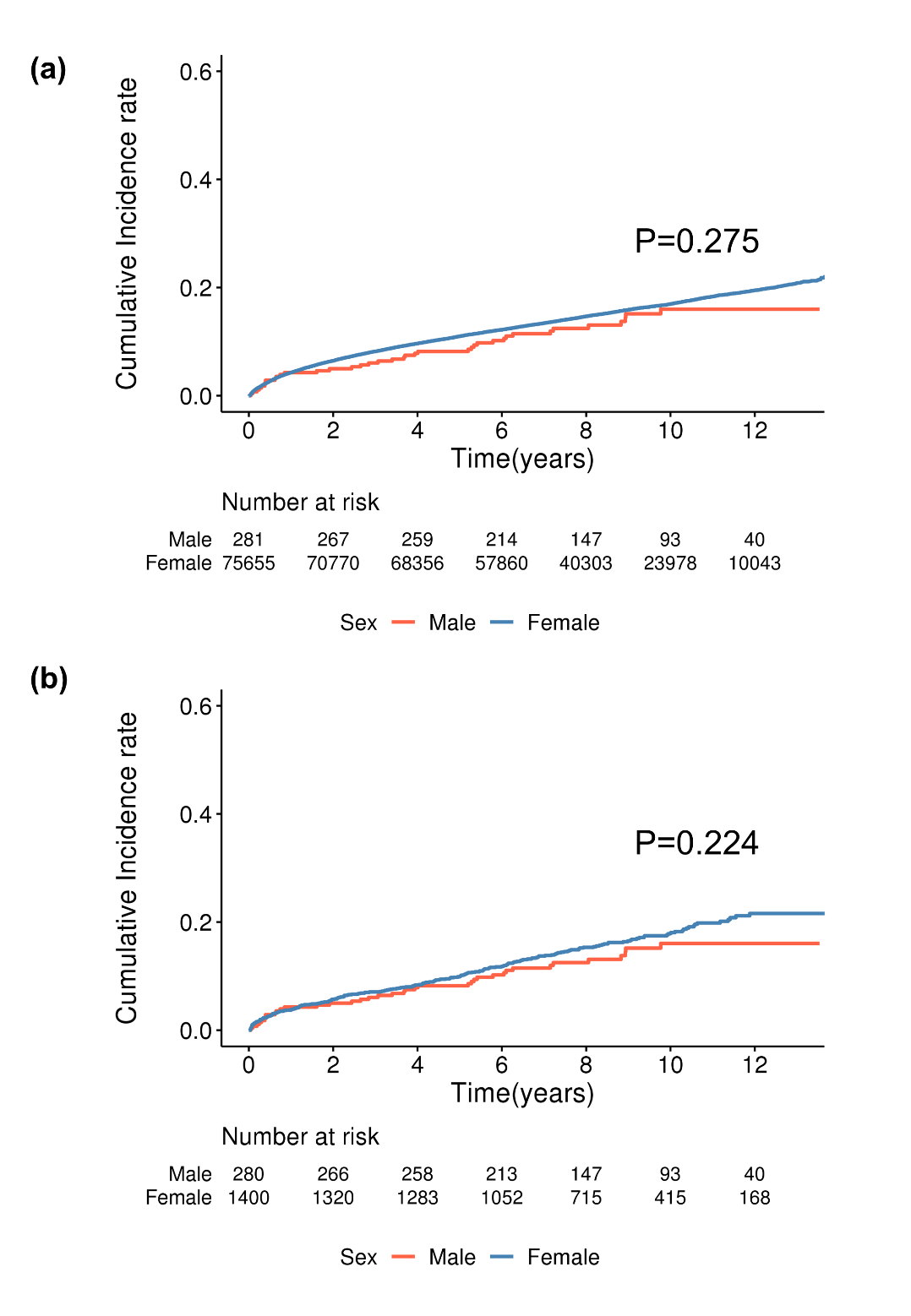
**

**Supplementary Figure 3. Kaplan–Meier analysis of the incidence of sleep disorder in breast cancer patients according to gender (median follow up 8.33±3.22 years).** Before matching, there was no significant difference in the incidence of sleep disorder in women compared to men (a, male n=281, female n=75,655, p=0.090, log-rank test). Even after matching, there was no statistically significant difference in the incidence of sleep disorder between men and women (b, male n=280, female n=1,400, p=0.102, log-rank test).


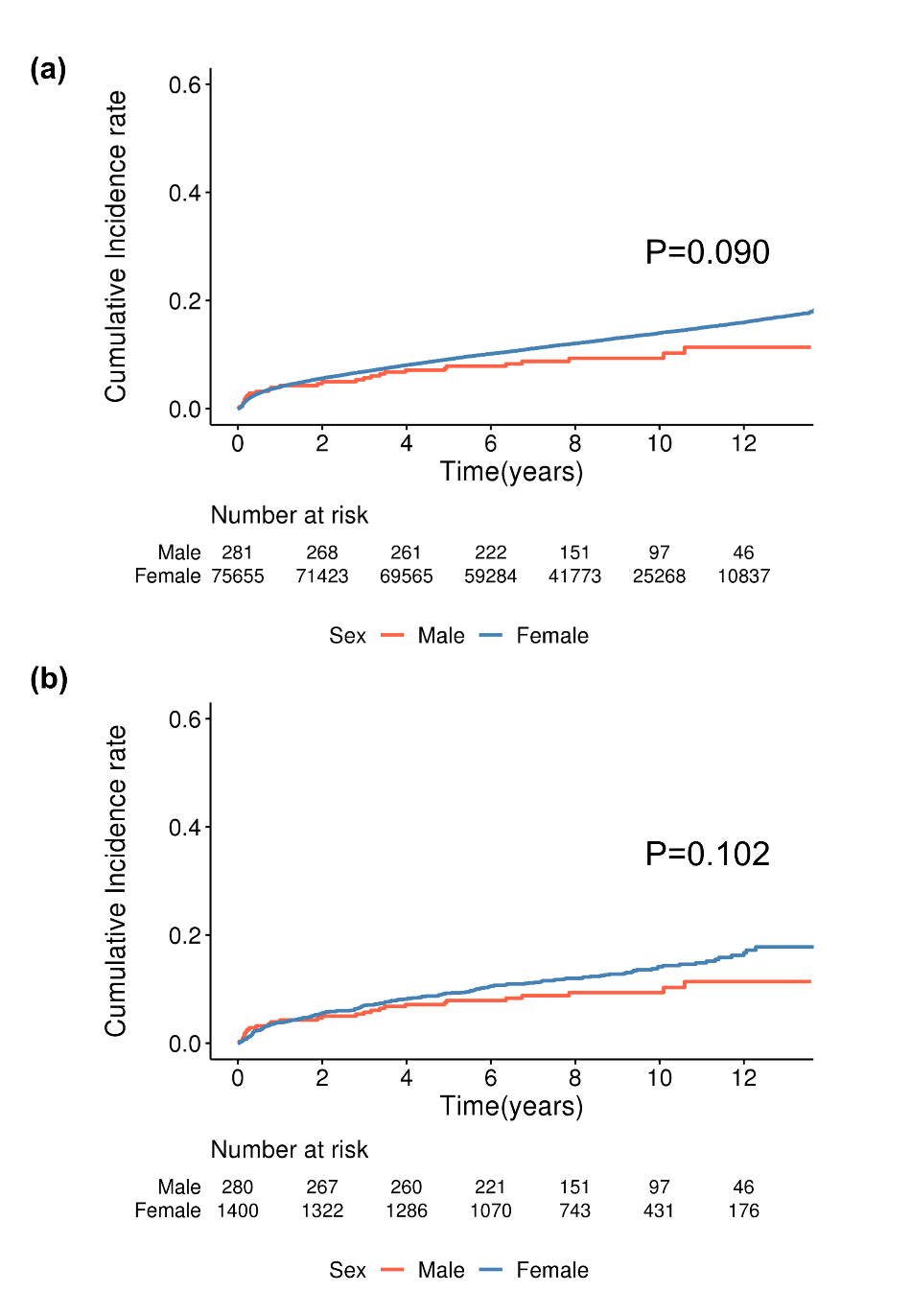


**Supplementary Table 1. Surgical codes for breast and axillary surgery**

| **Behavior code** | **ICD-10 Code** |
| --- | --- |
| N7133 | wide excision |
| N7134 | wide excision of axillary breast |
| N7135 | radical mastectomy including modified radical mastectomy and radical wide excision with axillary surgery |
| N7136 | wide excision with axillary surgery |
| N7137 | wide excision without axillary surgery |
| N7138 | total mastectomy with axillary surgery |
| N7139 | total mastectomy without axillary surgery |

**Supplementary Table 2. Drug prescription codes**

| **Drugs** | ***Codes** |
| --- | --- |
| **Tamoxifen** | 234501ATB, 234502ATB |
| **Aromatase inhibitor** |  |
| Letrozole | 182201ATB |
| Anastrozole | 109001ATB |
| Exemestane | 358401ATB |
| **GnRH analogues** | 202BIJ, 167201BIJ, 182602BIJ, 182604BIJ, 182611BIJ, 220603BIJ 244902BIJ 244901BIJ 244930BIJ 467501BIJ 467502BIJ |
| **Taxane** | 148301BIJ, 148302BIJ, 148303BIJ, 148304BIJ, 148305BIJ, 148306BIJ, 148309BIJ, 148310BIJ, 148338BIJ, 148339BIJ, 148340BIJ, 148341BIJ, 148342BIJ, 148344BIJ, 148345BIJ, 148346BIJ, 148348BIJ, 148349BIJ, 148350BIJ, 148351BIJ |
| **Doxorubicin** | 149401BIJ, 149402BIJ, 149403BIJ, 149404BIJ, 149405BIJ, 149406BIJ, 149430BIJ, 149431BIJ, 149432BIJ, 149433BIJ, 149434BIJ, 149435BIJ |
| **Cyclophosphamide** | 139001ATB, 139003BIJ, 139004BIJ, 139005BIJ |
| **Fluorouracil** | 161401BIJ, 161402BIJ, 161404BIJ, 161430BIJ, 161431BIJ, 161432BIJ |
| **Methotrexate** | 192101ATB, 192102BIJ, 192103BIJ, 192104BIJ, 192105BIJ, 192107ATB, 192107BIJ, 192108BIJ, 192109BIJ, 192110BIJ, 192111BIJ, 192132BIJ, 192134BIJ, 192136BIJ, 192138BIJ, 192139BIJ, 192140BIJ, 192141BIJ, 192142BIJ, 192143BIJ, 192144BIJ |
| **Carboplatin** | 123703BIJ, 123730BIJ, 123701BIJ, 123731BIJ, 123702BIJ, 123732BIJ, 123707BIJ, 123733BIJ, 123708BIJ, 123735BIJ, 123704BIJ, 123734BIJ, 123706BIJ, 123736BIJ |
| **Trastuzumab** | 242802BIJ, 242801BIJ, 626001BIJ, 626002BIJ, 242803BIJ, 242830BIJ |
| **Pertuzumab** | 624601BIJ |

Drug claim codes from the National Health Insurance Service

GnRh, gonadotropin releasing hormone

**Supplementary Table 3. Diagnostic codes for mental illness**

| **Classification** | **Diagnosis** | **ICD-10 code** |
| --- | --- | --- |
| **Anxiety disorder** | Agoraphobia | F40.0 |
|  | Social phobias | F40.1 |
|  | Specific (isolated) phobias | F40.2 |
|  | Other phobic anxiety disorders | F40.8 |
|  | Phobic anxiety disorder, unspecified | F40.9 |
|  | Panic disorder [episodic paroxysmal anxiety] | F41.0 |
|  | Generalized anxiety disorder | F41.1 |
|  | Mixed anxiety and depressive disorder | F41.2 |
|  | Anxiety depression(mild or not persistent) | F41.2 |
|  | Other mixed anxiety disorders | F41.3 |
|  | Other specified anxiety disorders | F41.8 |
|  | Anxiety disorder, unspecified | F41.9 |
| **Depressive disorder** | Mild depressive episode | F32.0 |
|  | Moderate depressive episode | F32.1 |
|  | Severe depressive episode without psychotic symptoms | F32.2 |
|  | Severe depressive episode with psychotic symptoms | F32.3 |
|  | Other depressive episodes | F32.8 |
|  | Depressive episode, unspecified | F32.9 |
|  | Recurrent depressive disorder, current episode mild | F33.0 |
|  | Recurrent depressive disorder, current episode moderate | F33.1 |
|  | Recurrent depressive disorder, current episode severe without psychotic symptoms | F33.2 |
|  | Recurrent depressive disorder, current episode severe with psychotic symptoms | F33.3 |
|  | Recurrent depressive disorder, currently in remission | F33.4 |
|  | Other recurrent depressive disorders | F33.8 |
|  | Recurrent depressive disorder, unspecified | F33.9 |
|  | Dysthymia | F34.1 |
|  | Other persistent mood[affective] disorders | F34.8 |
|  | Persistent mood[affective] disorder, unspecified | F34.9 |
|  | Other single mood[affective] disorders | F38.0 |
|  | Other recurrent mood[affective] disorders | F38.1 |
|  | Other specified mood [affective] disorders | F38.8 |
|  | Unspecified mood[affective] disorder | F39 |
| **Sleep disorder** | Nonorganic insomnia | F51.0 |
|  | Nonorganic hypersomnia | F51.1 |
|  | Nonorganic disorder of the sleep-wake schedule | F51.2 |
|  | Sleepwalking [somnambulism] | F51.3 |
|  | Sleep terrors [night terrors] | F51.4 |
|  | Nightmares | F51.5 |
|  | Other nonorganic sleep disorders | F51.8 |
|  | Nonorganic sleep disorder, unspecified | F51.9 |
|  | Disorders of initiating and maintaining sleep [insomnias] | G47.0 |
|  | Disorders of excessive somnolence [hypersomnias] | G47.1 |
|  | Disorders of the sleep-wake schedule | G47.2 |
|  | Obstructive sleep apnoea | G47.30 |
|  | Central sleep apnoea | G47.31 |
|  | Mixed sleep apnoea | G47.32 |
|  | Other sleep apnoea | G47.38 |
|  | Narcolepsy and cataplexy | G47.4 |
|  | Other sleep disorders | G47.8 |
|  | Personal history of unhealthy sleep-wake schedule | Z91.3 |

**Supplementary Table 4. Charlson Comorbidity Index**

| **Comorbidity** | **ICD-10 code** | **Updated Weight** |
| --- | --- | --- |
| Myocardial infarction | I21.x, I22.x, I25.2 | 0 |
| Congestive heart failure | I09.9, I11.0, I13.0, I13.2, I25.5, I42.0, I42.5-I42.9, I43.x, I50.x, P29.0 | 2 |
| Peripheral vascular disease | I70.x, I71.x, I73.1, I73.8, I73.9, I77.1, I79.0, I79.2, K55.1, K55.8, K55.9, Z95.8, Z95.9 | 0 |
| Cerebrovascular disease | G45.x, G46.x, H34.0, I60.x, I69.x | 0 |
| Dementia | F00.x-F03.x, F05.1, G30.x, G31.1 | 2 |
| Chronic pulmonary disease | I27.8, I27.9, J40.x-J47.x, J60.x-J67.x, J68.4, J70.1, J70.3 | 1 |
| Rheumatologic disease | M05.x, M06.x, M31.5, M32.x, M34.x, M35.1, M35.3, M36.0 | 1 |
| Peptic ulcer disease | K25.x-K28.x | 0 |
| Mild liver disease | B18.x, K70.0-K70.3, K70.9, K71.3-71.5, K71.7, K73.x, K74.x, K76.0, K76.2-K76.4, K76.8, K76.9, KZ94.4 | 2 |
| Diabetes without chronic complication | E10.0, E10.1, E10.6, E10.8, E10.9, E11.0, E11.1, E11.6, E11.8, E11.9, E12.0, E12.1, E12.6, E12.8, E12.9, E13.0, E13.1, E13.6, E13.8, E13.9, E14.0, E14.1, E14.8, E14.9 | 2 |
| Diabetes with chronic complication | E10.2-E10.5, E10.7, E11.2-E11.5, E11.7, E12.2-E12.5, E12.7, E13.2-E13.5, E13.7, E14.2-E14.5, E14.7 | 1 |
| Hemiplegia or paraplegia | G04.1, G11.4, G80.1, G80.2, G81.x, G82.x, G83.0-G83.4, G83.9 | 2 |
| Renal disease | I12.0, I13.1, N03.2-N03.7, N05.2-N05.7, N18.x, N19.x, N25.0, Z49.0-Z49..2, Z94.0, Z99.2 | 1 |
| Any malignancy including leukemia and lymphoma | There are no eligible patients in this study | 2 |
| Moderate or severe liver disease | I185.0, I85.9, I86.4, I98.2, K70.4, K71.1, K72.1, K72.9, K76.5, K76.6, K76.7 | 4 |
| Metastatic solid tumor | There are no eligible patients in this study | 6 |
| Acquired immune deficiency syndrome/human immune deficiency virus | B20.x-B22.x, B24.x | 4 |

**Supplementary Table 5. Risk of developing anxiety disorder from analyses using Cox proportional hazard models**

|  | **Before matching** | | **After matching** | | **Before matching** | | **After matching** | | |
| --- | --- | --- | --- | --- | --- | --- | --- | --- | --- |
|  | **Univariate analysis** | | **Univariate analysis** | | **Multivariate analysis** | | **Multivariate analysis** | | |
|  | **HR (95% CIs)** | ***P* value** | **HR (95% CIs)** | ***P* value** | **HR (95% CIs)** | ***P* value** | | **HR (95% CIs)** | ***P* value** |
| **Age (per 10-year)** | 1.090 (1.075-1.106) | <0.001 | 1.131 (1.040-1.231) | 0.004 | 1.086 (1.070-1.103) | <0.001 | | 1.160 (1.058-1.271) | 0.002 |
| **Gender** |  | 0.008 |  | 0.025 |  | 0.001 | |  | 0.023 |
| **Male** | Reference |  | Reference |  | Reference |  | | Reference |  |
| **Female** | 1.557 (1.123-2.158) |  | 1.491 (1.052-2.113) |  | 1.728 (1.246-2.396) |  | | 1.498 (1.057-2.123) |  |
| **CCI (Weight number)** | 1.051 (1.044-1.058) | <0.001 | 1.030 (0.989-1.072) | 0.154 | 1.039 (1.032-1.046) | <0.001 | | 1.017 (0.975-1.060) | 0.438 |
| **Chemotherapy** |  | <0.001 |  | 0.325 |  | <0.001 | |  | 0.183 |
| **Not done** | Reference |  | Reference |  | Reference |  | | Reference |  |
| **Done** | 1.308 (1.265-1.353) |  | 1.124 (0.891-1.419) |  | 1.318 (1.272-1.365) |  | | 1.179 (0.925-1.504) |  |
| **Endocrine therapy** |  | <0.001 |  | 0.115 |  | 0.001 | |  | 0.045 |
| **Not done** | Reference |  | Reference |  | Reference |  | | Reference |  |
| **Done** | 0.905 (0.872-0.938) |  | 1.453 (0.913-2.313) |  | 0.941 (0.907-0.977) |  | | 1.628 (1.012-2.621) |  |
| **HER2-target therapy** |  | <0.001 |  | 0.662 |  | 0.605 | |  | 0.798 |
| **Not done** | Reference |  | Reference |  | Reference |  | | Reference |  |
| **Done** | 1.146 (1.094-1.202) |  | 0.887 (0.519-1.518) |  | 0.987 (0.939-1.037) |  | | 0.931 (0.537-1.613) |  |

CCI, Charlson Comorbidity index

**Supplementary Table 6. Risk of developing depression disorder from analyses using Cox proportional hazard models**

|  | **Before matching** | | **After matching** | | **Before matching** | | **After matching** | | |
| --- | --- | --- | --- | --- | --- | --- | --- | --- | --- |
|  | **Univariate analysis** | | **Univariate analysis** | | **Multivariate analysis** | | **Multivariate analysis** | | |
|  | **HR (95% CIs)** | ***P* value** | **HR (95% CIs)** | ***P* value** | **HR (95% CIs)** | ***P* value** | | **HR (95% CIs)** | ***P* value** |
| **Age (per 10-year)** | 1.098 (1.081-1.115) | <0.001 | 1.186 (1.085-1.296) | 0.001 | 1.087 (1.069-1.105) | <0.001 | | 1.207 (1.095-1.330) | 0.001 |
| **Gender** |  | 0.276 |  | 0.225 |  | 0.064 | |  | 0.213 |
| **Male** | Reference |  | Reference |  | Reference |  | | Reference |  |
| **Female** | 1.557 (1.123-2.158) |  |  |  |  |  | |  |  |
| **CCI (Weight number)** | 1.054 (1.046-1.062) | <0.001 | 1.065 (1.023-1.108) | 0.002 | 1.044 (1.036-1.052) | <0.001 | | 1.048 (1.006-1.092) | 0.026 |
| **Chemotherapy** |  | <0.001 |  | 0.433 |  | <0.001 | |  | 0.192 |
| **Not done** | Reference |  | Reference |  | Reference |  | | Reference |  |
| **Done** | 1.140 (1.099-1.182) |  | 1.103 (0.863-1.408) |  | 1.161 (1.117-1.207) |  | | 1.185 (0.918-1.529) |  |
| **Endocrine therapy** |  | 0.441 |  | 0.058 |  | 0.045 | |  | 0.014 |
| **Not done** | Reference |  | Reference |  | Reference |  | | Reference |  |
| **Done** | 1.016 (0.975-1.059) |  | 1.654 (0.983-2.785) |  | 1.044 (1.001-1.088) |  | | 1.954 (1.148-3.325) |  |
| **HER2-target therapy** |  | 0.113 |  | 0.301 |  | 0.238 | |  | 0.469 |
| **Not done** | Reference |  | Reference |  | Reference |  | | Reference |  |
| **Done** | 1.044 (0.990-1.101) |  | 0.727 (0.398-1.330) |  | 0.967 (0.914-1.023) |  | | 0.796 (0.430-1.474) |  |

CCI, Charlson Comorbidity index

**Supplementary Table 7. Risk of developing sleep disorder from analyses using Cox proportional hazard models**

|  | **Before matching** | | **After matching** | | **Before matching** | | **After matching** | | |
| --- | --- | --- | --- | --- | --- | --- | --- | --- | --- |
|  | **Univariate analysis** | | **Univariate analysis** | | **Multivariate analysis** | | **Multivariate analysis** | | |
|  | **HR (95% CIs)** | ***P* value** | **HR (95% CIs)** | ***P* value** | **HR (95% CIs)** | ***P* value** | | **HR (95% CIs)** | ***P* value** |
| **Age (per 10-year)** | 1.072 (1.053-1.091) | <0.001 | 1.055 (0.956-1.164) | 0.290 | 1.075 (1.056-1.095) | <0.001 | | 1.057 (0.950-1.175) | 0.309 |
| **Gender** |  | 0.092 |  | 0.105 |  | 0.028 | |  | 0.102 |
| **Male** | Reference |  | Reference |  | Reference |  | | Reference |  |
| **Female** | 1.384 (0.949-2.018) |  | 1.397 (0.933-2.093) |  | 1.526 (1.046-2.227) |  | | 1.401 (0.936-2.099) |  |
| **CCI (Weight number)** | 1.039 (1.030-1.047) | <0.001 | 1.083 (1.036-1.132) | 0.001 | 1.028 (1.019-1.037) | <0.001 | | 1.081 (1.033-1.131_ | 0.001 |
| **Chemotherapy** |  | <0.001 |  | 0.156 |  | <0.001 | |  | 0.318 |
| **Not done** | Reference |  | Reference |  | Reference |  | | Reference |  |
| **Done** | 1.289 (1.238-1.342) |  | 1.224 (0.926-1.617) |  | 1.290 (1.236-1.347) |  | | 1.162 (0.866-1.559) |  |
| **Endocrine therapy** |  | 0.016 |  | 0.42 |  | <0.001 | |  | 0.444 |
| **Not done** | Reference |  | Reference |  | Reference |  | | Reference |  |
| **Done** | 1.058 (1.011-1.108) |  | 1.241 (0.734-2.099) |  | 1.108 (1.058-1.161) |  | | 1.235 (0.720-2.117) |  |
| **HER2-target therapy** |  | <0.001 |  | 0.057 |  | 0.009 | |  | 0.053 |
| **Not done** | Reference |  | Reference |  | Reference |  | | Reference |  |
| **Done** | 1.205 (1.140-1.275) |  | 1.618 (0.985-2.657) |  | 1.083 (1.020-1.149) |  | | 1.664 (0.993-2.787) |  |

CCI, Charlson Comorbidity index
